# The sweet and sour of serological glycoprotein tumor biomarker quantification

**DOI:** 10.1186/1741-7015-11-31

**Published:** 2013-02-07

**Authors:** Uros Kuzmanov, Hari Kosanam, Eleftherios P Diamandis

**Affiliations:** 1 Department of Laboratory Medicine and Pathobiology, University of Toronto, Toronto, 6th floor, 60 Murray Street, Box 32, Toronto, ON M5T 3L9, Canada; 2 Department of Pathology and Laboratory Medicine, Mount Sinai Hospital, 6th Floor, 60 Murray Street, Box 32, Toronto, ON M5T 3L9, Canada; 3 Department of Clinical Biochemistry, University Health Network, 6th Floor, 60 Murray Street, Box 32, Toronto, ON M5T 3L9, Canada

**Keywords:** glycobiomarker, glycopeptide, lectin, lectin ELISA, mass spectrometry, N-glycosylation, ovarian cancer, sialic acid.

## Abstract

Aberrant and dysregulated protein glycosylation is a well-established event in the process of oncogenesis and cancer progression. Years of study on the glycobiology of cancer have been focused on the development of clinically viable diagnostic applications of this knowledge. However, for a number of reasons, there has been only sparse and varied success. The causes of this range from technical to biological issues that arise when studying protein glycosylation and attempting to apply it to practical applications. This review focuses on the pitfalls, advances, and future directions to be taken in the development of clinically applicable quantitative assays using glycan moieties from serum-based proteins as analytes. Topics covered include the development and progress of applications of lectins, mass spectrometry, and other technologies towards this purpose. Slowly but surely, novel applications of established and development of new technologies will eventually provide us with the tools to reach the ultimate goal of quantification of the full scope of heterogeneity associated with the glycosylation of biomarker candidate glycoproteins in a clinically applicable fashion.

## Protein glycosylation

**Figure 1:**
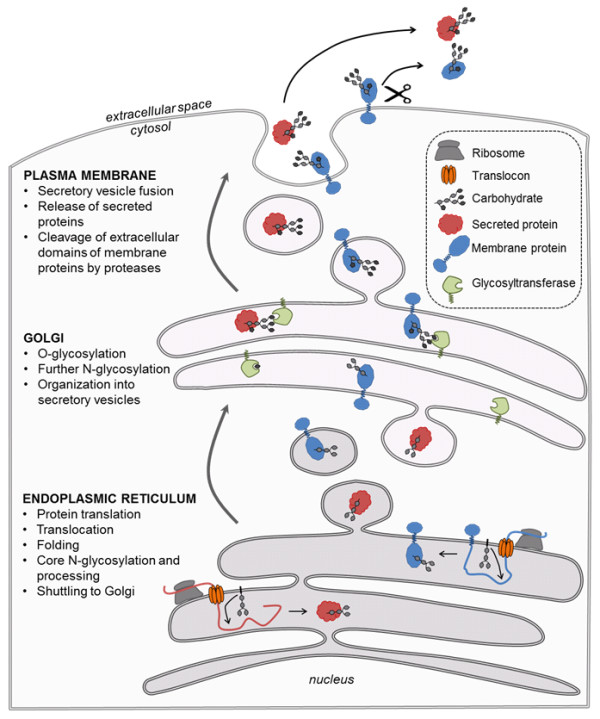
**Life span of glycoproteins from translation to circulation**. The translation of signal peptide-containing membrane and secreted protein occurs on the surface of the endoplasmic reticulum (ER), with the growing peptide chain being shuttled through the translocon complex into the lumen of the ER. In the ER lumen, core N-glycosylation of accessible N-X-S/T sites is performed by the oligosaccharide transferase component of the translocon complex while the nascent protein is being translated and folded. Following the completion of translation, folding, and core glycan processing, the protein is shuttled to the Golgi apparatus, where further N-glycosylation and O-glycosylation are performed by different glycosyltransferases. In the Golgi, glycoproteins are packaged into secretory vesicles bound for fusion with the plasma membrane, where the secreted proteins are released into the extracellular space and membrane proteins are presented on the surface of the cell, making them accessible for cleavage and release by proteolytic enzymes. Once in the extracellular space, these glycoproteins can then enter the circulation.

It is a well-established concept that gene expression and protein expression are not the sole factors responsible for phenotype determination. The discovery of the varying roles of post-translational modifications (PTMs) of proteins has identified another level at which functional information is stored. Of the more than 200 different types of protein PTMs, glycosylation is a frequently occurring and particularly important one [1-4]. Glycosylation has been shown to have an important role in a number of physiological processes, including protein folding and trafficking, cell-cell and cell-matrix interaction, cellular differentiation, fertilization, and the immune response [5-9]. Approximately half of all mammalian proteins are glycosylated, with an estimated 3,000 different glycan structures recorded (not including all variants resulting from differences in glycan linkages and anomers), which can vary to a large degree, based on differences in tissue, cell type, and disease state [10,11]. It is estimated that 250 to 500 genes are involved in the protein glycosylation process [12]. Carbohydrate molecules on proteins can be attached to asparagine residues within the N-X-S/T consensus sequence when X is not a proline (N-glycosylation), or to serine or threonine residues (O-glycosylation). This occurs during or after translation as the nascent protein is shuttled through the endoplasmic reticulum (ER) and subsequent organelles in the classical secretory pathway (Figure 1). However, glycosylation is not a template-based process such as DNA, RNA, or protein synthesis, but is rather based on the balance achieved by the expression and activity levels of the different glycan attachment and processing enzymes involved in trimming and addition of monosaccharides, and on the availability of precursor monosaccharide molecules, which in turn is dependent on nutrient resources and expression of other metabolic enzymes responsible for their synthesis and interconversion [7,8,13]. This greatly increases the complexity of the protein glycosylation process, resulting in extensive molecular microheterogeneity of glycoproteins, and thus the requirement for a specialized set of tools for their study.

## Glycosylation in cancer

Since the initial observation in 1969 showing that membrane glycoproteins of higher molecular weight were present in transformed mouse fibroblasts compared with their normal counterparts [14,15], aberrant glycosylation patterns have been established as a common characteristic of oncologic malignancies. These patterns have been observed in almost all types of experimental and human cancers. Even under non-malignant conditions, individual glycoproteins are produced in a number of different glycoforms [16]. The differences in these forms can arise from differential occupancy of glycosylation sites or variability in attached glycan structures. This allows for great heterogeneity in glycosylation of single proteins even under normal physiological conditions. However, under normal physiologic conditions, the distribution of these glycoforms is stable and reproducible. Once malignant transformation occurs, when underexpression, overexpression, or neoexpression of glycan moieties can occur, this balance is disturbed, and can expand the degree of pre-existing microheterogeneity of individual proteins [17]. In tumors, the changes in glycan structures most often arise from disturbances in the expression and activity levels of different glycosyltransferases and glycosidases along the secretory pathway, in the ER and Golgi of cancer cells [18-22]. This can lead to changes in the structures of both N- and O- linked glycans. For example, increased activity or expression of N-acetylglucosaminyltransferase V (MGAT5) has been shown in a number of tumors, resulting in increased glycan branching on proteins and increased tumor growth and metastasis [23-27]. Alterations in terminal glycan residues can also occur during malignancy, which is often the case with the upregulation of different sialyltransferase enzymes in tumors [28-33]. However, it must be noted that altered glycosylation does not only occur on proteins produced by the tumor itself, but may reflect the host's response to the disease. In patients with cancer, acute-phase proteins and IgGs have been shown to have glycosylation patterns distinct from those found under normal physiological conditions [18]. Therefore, the detection and quantification of the disturbances in protein glycosylation can aid in the screening and diagnosis of virtually all cancer types.

## Glycoprotein cancer biomarkers

Some of the oldest and most common clinically utilized serological biomarkers for cancer diagnosis and monitoring of malignant progression are glycoproteins. Some of these include prominent glycoprotein biomarkers that are widely monitored in patients with prostate cancer (prostate-specific antigen (PSA)), ovarian cancer (carcinoma antigen (CA)125, mucin 16), colon cancer (carcinoembryonic antigen (CEA)), and non-seminomatous testicular carcinoma (human chorionic gonadotropin β-subunit (hCG-β)) (Table 1). Although all of these proteins have been shown to have aberrant glycosylation patterns in malignancy [29-37], only their total protein levels are clinically monitored. Simultaneous measurement of their different glycoforms might increase the diagnostic potential of these molecules. For two other common tests, alpha-fetoprotein (AFP) for hepatocellular carcinoma and CA15-3 (mucin 1 epitope) for breast cancer, specific glycan structures on these proteins are monitored, as discussed below.

**Table 1:** List of common serological tumor markers in clinical use that contain a glycan component^a^

Biomarker	Type of detection	Cancer type(s)	Clinical applications	References
**AFP**	Protein and core fucosylation (for AFP-L3)	Germ-cell hepatoma; non-seminomatous testicular carcinoma	Diagnosis, staging. detecting recurrence, monitoring therapy	[153,154]

**hCG**	Protein alone	Testicular	Diagnosis;staging; detecting recurrence; monitoring therapy	[154,155]

**CA125**	Protein alone	Ovarian	Prognosis; detecting recurrence; monitoring therapy	[154,156]

**CA15-3**	Sialylated O-glycan on MUC1	Breast	Monitoring therapy	[157-159]

**CA19-9**	SLe on mucin glycoproteins and gangliosidesa	Pancreatic	Monitoring therapy	[160,161]

**CEA**	Protein alone	Colon	Detecting recurrence; monitoring therapy	[154,157,161]

**HER2**	Protein alone	Breast	Therapy selection	[157,162,163]

**PSA**	Protein alone	Prostate	Screening; diagnosis (with digital rectal examination)	[154,164]

**Thyroglobulin**	Protein alone	Thyroid	Monitoring	[165,166]

**CA27-29**	MUC1 protein alone	Breast	Monitoring	[161,167]

**^a^**Modified and adapted from Kulasingam and Diamandis [168]. AFP, alpha-fetoprotein; CA, carcinoma antigen 15-3; CA 19-9, carbohydrate antigen 19-9; CEA, carcinoembryonic antigen; HER, herceptin; hCG, human chorionic gonadotropin; MRM, multiple reaction monitoring; PSA, prostate-specific antigen; SLe, sialyl Lewis antigen.

Some of the most widely used discovery platforms for the identification of novel glycobiomarkers have been previously reviewed [17,38-40]. The methods used for the characterization and analysis of glycan-based cancer biomarkers currently used clinically and for others in earlier stages of development have also been previously reviewed by Adamczyk *et al*. [41]. In the present review, we focus on the currently available and potential future techniques that can be used for the quantification of glycoprotein biomarkers in biological fluid or serum patient samples.

There are three general approaches, using a variety of techniques, by which glycoproteins or carbohydrate epitopes can be quantified. The most commonly used approach involves the measurement of total levels of a given glycoprotein biomarker. This usually involves the production of monoclonal antibodies against a given glycoprotein, facilitating the development of an assay capable of quantifying total protein levels in the biological fluid of interest. This is the case with PSA, CA125, hCG-b, and CEA quantification (Table 1). However, this type of methodology is not capable of detecting the changes occurring in the glycosylation patterns of the target glycoprotein as a result of malignant transformation, thus missing out on another level of information that could lead to improved diagnosis and monitoring of disease. Therefore, even though a glycoprotein is being measured, its glycan moiety is completely ignored.

Another approach involves the detection and quantification of a particular glycan structure shown to be associated with cancer, such as the antibody-based measurement of the blood group antigen Lewis^a ^in the CA19-9 assay [42]. This type of approach does not yield any information about the identity or quantity of the glycoprotein with the particular carbohydrate epitope, thus also does not include the full scope of information, which could lead to improved diagnosis, especially if the protein is produced directly by the tumor.

The third, most rarely used, and most difficult type of approach to develop allows for detection and quantification of both total protein levels and associated glycan structure(s), such as the measurement of the core-fucosylated species of AFP in hepatocellular carcinoma [43,44]. This type of assay can yield the most information and overcomes the weaknesses of the other two approaches mentioned above. Therefore, the development of such a method would have the most diagnostic benefit.

## The potential and the pitfalls

In the past decade or so, there have been significant advancements in the characterization of the glycosylation patterns of individual proteins and in the identification of glycoproteins in a number of complex biological fluids. This has occurred mostly through the development and refinement of mass spectrometry techniques and equipment, which, when used in concert with the traditional methods used for characterization of protein glycosylation, can provide a powerful complement of tools to tackle the problem of fully understanding the complexity and heterogeneity associated with protein glycosylation and applying the gained knowledge in a clinical setting. However, there has been limited progress in tapping the full potential of glycobiomarkers and their dual nature in order to develop an assay capable of simultaneously delivering information on the absolute quantity of the protein and of its associated glycan structures in complex matrices, such as serum, which is the preferred sample type for high-throughput clinical analysis.

**Figure 2:**
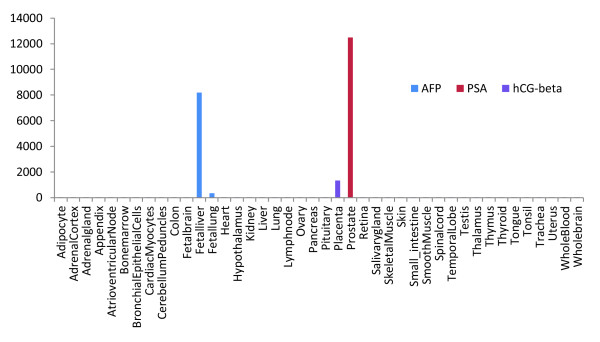
**Gene expression of alpha-fetoprotein (AFP), beta-human chorionic gonadotropin (beta HCG), and prostate-specific antigen (PSA) by tissue**. Figure adapted and modified from the BioGPS Application [151], using the HG_U133A/GNF1H Gene Atlas [152].

Some of the best and most widely recognized cancer biomarkers are highly tissue-specific, such as PSA for prostate tissue, hCG for the placenta, and AFP for the developing fetus (Figure 2). Using such markers malignant transformation of cells in a single organ causing the overexpression or neoexpression of a protein can be detected and monitored more reliably and earlier in the progression of the disease, compared with a protein expressed ubiquitously or in multiple tissues. However, proteins with such characteristics are quite rare. Considering that glycosylation patterns of the same protein can differ both between tissues and between normal and transformed cells, the capability of detecting and quantifying these differences could confer tissue-/tumor-specific profiles on a large number of glycoproteins. The ability to perform such a task reliably, and in a routine fashion, could greatly expand the field of potential biomarkers and the chances of their application in the clinical setting.

However, there is a series of technical and biological obstacles to developing quantitative assays that reflect the full picture of the status of a glycoprotein biomarker. The majority of challenges preventing reliable, clinically applicable binary measurement of glycoprotein biomarkers are of a technical nature. More specifically, there is only a very limited set of tools capable of performing this task, each with its own set of associated limitations and difficulties. Currently, the options for concurrent quantification of a protein and its associated glycans are limited to a combination of antibody-mediated protein capture and detection with glycan-specific antibodies, lectins, or mass spectrometry. The advancement of these approaches is hindered by the absence of a suitable recombinant technology capable of reliable and convenient production of glycoproteins with the desired glycan structures, which would allow for more convenient and detailed studies. However, because protein glycosylation is not a template-driven linear sequence-based process, such as DNA or protein synthesis, a suitable solution to this problem does not seem to be on the horizon, even though some advancements have been made [45]. Owing to the large number of combinations of branched oligosaccharide structures that can be created from available monosaccharides in eukaryotic cells, and especially cancer cells, where target protein production and normal glycosylation processes are greatly disturbed, the staggering glycan microheterogeneity can significantly impede the precise binary measurement of individual glycobiomarkers [46]. That is why the majority of proteins for which the development of these types of assays has been attempted are high-abundance proteins themselves (for example, transferrin, haptoglobin, IgGs, and alpha-1-acid glycoproteins). Therefore, a quantitative detection system encompassing the heterogeneity of glycan structures of a single protein in a single output holds great potential for bringing the use of more glycobiomarkers to a respectable (clinically testable) level.

The majority of the top 22 high-abundance plasma proteins, which account for 99% of protein content in serum, are glycoproteins [47]. These include such proteins as the Ig family members, haptoglobin, antitrypsin, and transferrin, among others. However, the majority of potential biomarkers are found at significantly (several orders of magnitude) lower levels in the serum. Taking into consideration that a specific glycan profile on one protein might indicate a malignant condition, but the same profile on another protein (for example, one of the high-abundance proteins) might not, the specificity of detection of low-concentration serum glycoproteins by lectins or even glycan-specific antibodies can be hindered by high background levels of contaminating high-abundance glycoproteins. Thus, these methods of detection lag far behind the gold standard (sandwich ELISA) in sensitivity, especially when taking into account that only a subset of the total population of the target protein is being measured.

Therefore, in this review, we focus on the technologies with the capability or a strong potential for binary (protein and carbohydrate) measurement of glycoprotein cancer biomarkers in serum, and describe the challenges associated with the different approaches.

## Lectin-based methods

The existence of lectins has been known for over 100 years, since the discovery of ricin by Stillmark in 1888 [48]. However, wider application in research did not occur until the early 1970s [49,50]. Lectins are proteins with a proven affinity and selectivity for specific carbohydrate structures, to which they can bind in a reversible fashion. Lectins can recognize carbohydrates conjugated to proteins and lipids, or free monosaccharides or polysaccharides. In excess of 500 lectins have been discovered, mostly of plant origin, and over 100 are commercially available [48]. They have been used in a wide variety of technical formats, including lectin blots, immunohistochemistry, liquid chromatography, and lectin microarrays. Despite extensive characterization and the many years of experience with lectin research, there are only a few applications in which lectins have been used in a clinically applicable high-throughput fashion to detect and quantify serological biomarkers in cancer. Lectins are the oldest and most reliable tools for glycoprotein characterization, and are indispensible in any endeavor involving analysis of protein-associated glycans; however, the lectin journey from an analytical to a quantitative tool has been a long one, with many obstacles and few successes.

Enzyme-linked lectin approaches for detection of carbohydrates have been known and used for close to 3 decades [51,52]. These types of quantitative assays have been ported into a high-throughput multiwell plate format, similar to the common ELISA technique in which a protein of interest is captured and/or detected by an antibody, but with lectins taking over the antibody roles. Over the years, there have been several types of assays, which can be grouped together under the common name of 'enzyme-linked lectin assay (ELLA)'. In one format, serum or cell-bound proteins are non-specifically immobilized, and the global levels of a particular glycan structure are detected using a specific lectin. This has been performed on the sera of patients with squamous cell carcinoma of the uterine cervix by measuring the levels of the Thomsen-Friendenreich antigen (T-Ag) using the peanut agglutinin (PNA) lectin for detection [53]. The reactivity of a number of lectins to serum glycoproteins from patients with lung cancer was also measured using this general approach [54]. It has also been used extensively for detection and differentiation of a number of species of bacteria [55-57]. In another use of lectins in an ELLA-type approach, an immobilized lectin is used to capture all glycoconjugates with a particular glycan structure from a complex biological sample, and the presence and quantity of a particular protein is then determined by antibody detection. An example of this approach was a study detecting wheat germ agglutinin (WGA)-bound mucins in the serum of patients with pancreatic cancer [58]. However, this approach requires the target glycoprotein to account for a significantly large proportion of the total glycoprotein content in the sample, which is often not the case. Another, more desirable approach involves the antibody-based capture of a single protein and subsequent detection of associated glycan components by lectins. This approach has been used to measure sialylation of transferrin [59], fucosylation of PSA in patients with prostate cancer [60], sialylation of recombinant erythropoietin [61], WGA and ConA reactivity to p185 in the serum of patients with breast cancer [62], and fucosylation of haptoglobin in the serum of patients with pancreatic cancer [63].

It must be noted that the antibody-lectin sandwich approaches are plagued by a number of technical issues, which can be addressed with varying degrees of success. A major issue is the inherent glycosylation of the antibodies used to capture a specific glycoprotein, which can cause a non-specific background signal from lectin binding, often masking the signal from the glycoprotein of interest. This effect can be minimized by the enzymatic or chemical derivatization of the antibody-associated carbohydrates prior to use in the assay [59,64,65]. Another issue is the limited recognition range of any given lectin for a particular glycan structure, thereby preventing the detection of the full scope of the heterogeneity of glycosylation on any particular glycoprotein. Use of multiple lectins for detection in an array format can ameliorate this issue (see below). When considering serum as the analyte matrix, another significant source of background signal in this type of assay comes from the non-specific contamination by high-abundance glycoproteins. This often masks the signal from low-abundance glycoprotein analytes. This is not an issue when measuring other high-abundance serum glycoproteins, such as transferrin [59] or haptoglobin [63], as dilution of the serum sample can lower the background noise to a minimal level. For low-abundance glycoproteins, for which sample dilution is not an option, more rigorous washing and blocking steps are required [66].

The greatest success with use of lectins for the diagnosis of malignant conditions has been the discovery and quantification of the *Lens culinaris *agglutinin (LCA)-reactive species of alpha-fetoprotein (AFP-L3). This has been shown to improve the specificity for hepatocellular carcinoma (HCC) compared with total AFP levels, as the latter can be elevated in pregnancy, hepatitis, and liver cirrhosis [43,44,67,68]. However, in an ingenious departure from the ELLA-type approach, in which a lectin replaces an antibody in an ELISA format, the AFP-L3 test relies on the liquid-phase capture of AFP reactive to LCA, and subsequent measurement of bound and unbound portions of the protein by an ELISA for total AFP. Therefore, the lectin is not used for detection but for fractionation of the AFP glycoprotein populations in the patient serum, and the quantification is performed by a standard ELISA developed with antibodies recognizing peptide (non-glycosylated) epitopes. It is highly fortuitous, given the microheterogeneity associated with AFP glycosylation in HCC, that only the core fucosylation status of the single N-glycosylation site of AFP, as detected by LCA, is sufficient for successful diagnosis [69,70].

Over the past decade, a new role has been identified for lectins in the characterization and quantification of serum glycoproteins in malignant conditions. In a re-imagination of the ELLA approach, multiple lectins are now being used to simultaneously detect different carbohydrate structures on antibody-captured glycoproteins in a microarray format. Several groups have created methods in which an antibody is immobilized in an array format and lectins are used to measure glycosylation of the captured proteins [65,71-73]. The major advantage of this approach is the ability to detect a glycan profile of any given glycoprotein, and to compare it between different samples in a high-throughput fashion. Aberrant glycosylation patterns of mucins, carcinoembryonic antigen-related cell adhesion molecule, and alpha-1-beta glycoprotein in clinical samples from patients with pancreatic cancer have been detected using similar methods by different groups [74-76]. This type of approach goes a long way toward detecting the heterogeneity of glycan structures of individual glycoproteins, but at the core, it is only multiplexing of the ELLA method, with its associated restrictions, which has been known and applied with limited success over the past 3 decades.

## Mass spectrometry-based methods

Advancements in mass spectrometry (MS) have revolutionized the field of carbohydrate research, and led to the initiation of a large number of studies dealing with the identification, analysis, and quantification of glycoconjugates [17,77]. With regard to glycosylated proteins, these studies range from inspections of individual glycoproteins to elucidation of whole glycoproteomes. Toward these ends, MS has been coupled to a number of well-established, as well as novel technologies, dealing with chemical modification, chromatographic separation, and affinity purification of glycans to achieve the best results. These studies have been conducted on multiple MS platforms, including ion trap (IT), linear trap quadrupole (LTQ), time of flight (TOF), quadrupole/triple quadrupole (Q), Orbitrap, and Fourier transform ion cyclotron resonance (FTICR) mass analyzers [39]. As a result of its proven utility, MS analysis has become an almost absolute requirement for any study dealing with the identification and analysis of protein glycosylation. MS-based approaches for glycoprotein identification, analysis, and characterization have been reviewed extensively and in a number of publications [17,39,40,77,78]. Several major groups have focused on liquid chromatography (LC)-coupled MS methodologies for glycan analysis, using separation and enrichment of glycans by hydrophilic interaction liquid chromatography (HILIC), porous graphitized carbon (PGC), and reverse-phase (RP) liquid chromatography. Some examples include studies on HILIC for analysis of native and derivatized glycans [79-81]; the use of PGC for enrichment and separation of native glycans [82,83]; and the work of Alley *et al*. and Mechref using RP LC [84,85]. However, the quantification of glycoproteins and their associated glycans using MS techniques is at a nascent stage, with no clinical applications to date. Similar to the strategies for identifying and characterizing protein glycosylation, MS can also be used to quantify glycoproteins only or glycoprotein-associated glycans only, or to simultaneously measure both the quantity of the protein and its associated carbohydrate structure. These quantification strategies have followed the same trend as the established MS-based techniques for quantifying proteins. These can be further separated into label-based or label-free approaches. Most of the common labeling methods have involved stable isotopic labeling techniques, such as ^16^O/^18^O, ^12^C/^13^C, stable isotope labeling with amino acids in culture (SILAC), isobaric tags (iTRAQ), and isotope-coded affinity tags (ICAT) [39]. These strategies are regularly used for comparison and relative quantification of glycoprotein analytes between samples. Label-free approaches have included spectral counting, ion-intensity measurement, and multiple/selected reaction monitoring (MRM/SRM). However, as can be seen from the majority of the recent examples in literature shown below, all of these approaches, and their combinations, have been limited to quantification of glycoproteins that are highly purified in background matrices much less complex than serum or other biological fluids of interest or dealing with one of the high-abundance proteins.

Although routinely used for identification and characterization purposes, an established application of MS in the glycomics field is the quantification of carbohydrates released, chemically or enzymatically, from individual or multiple glycoproteins. MALDI-MS instrumentation has been shown to be invaluable for this type of approach. This platform was used by two different groups to quantitate sialylated glycans enzymatically released (PNGase F-treated) glycoproteins in a high-throughput fashion. For example, a MALDI-TOF-based methodology was developed for absolute and relative measurement of up to 34 major N-glycans released from (mostly high-abundance) serum proteins by optimization of glycan release conditions through development of novel detergent reagents [86]. The diagnostic and stage stratification potential of MS-based quantification of permethylated glycans from serum proteins of patients with breast cancer was shown by a study that was able to identify and quantify close to 50 different glycan structures [87]. Relative quantification of anthranilic acid-derivatized glycans enzymatically released from alpha-1-acid glycoprotein purified from serum in combination with linear discriminant analysis has been shown to have the potential to discriminate between normal individuals and patients with ovarian cancer and lymphoma [88]. Similar approaches have also led to the identification of serum haptoglobin glycans with diagnostic potential in lung cancer [89] and liver disease [90].

Quantification of proteins, including some glycoproteins, by MRM/SRM and LC-MS has been performed for a number of biological fluids [91-94]. Great advances have been made with approaches using immunoaffinity enrichment of peptides or proteins followed by MRM/SRM-based quantification, achieving levels of sensitivity applicable to the concentration range (ng/ml) at which low-abundance tumor biomarkers are found [95-99]. This type of methodology has also been used in combination with different types of glycan affinity enrichment strategies, thereby producing hybrid assays in which classic glycoprotein enrichment strategies are used for capture of specific glycoforms, and MS is used for detection and quantification of the protein in those subpopulations by monitoring the MS2 fragmentation of non-glycosylated tryptic peptides. One such example was the quantification of the phytohemagglutinin-L4 (L-PHA)-enriched fraction of tissue inhibitor of metalloproteinase 1 from the serum of patients with colorectal carcinoma and the supernatant of colon cancer cell lines [100,101]. A number of high-abundance serum proteins were quantified recently in the serum of patients with HCC by the same group using a similar approach of glycoprotein enrichment by lectin and quantification by MRM [102]. Also, a method for the measurement of total glycosylated and sialylated PSA has been recently developed, in which periodate-oxidized PSA tryptic glycopeptides are captured using immobilized hydrazide, released by PNGase F, and quantified by MRM using a triple quadrupole LC-MS [103]. However, it must be noted that these types of studies do not exploit the full potential of MS in detection of the heterogeneity of glycan structures associated with any given glycoprotein, but rather use this technology solely for protein quantification, which could be performed more conveniently and reliably by classic methods such as ELISA.

**Figure 3:**
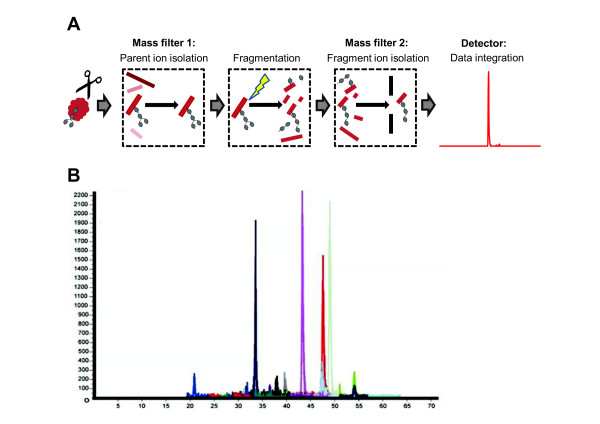
**Glycopeptide MRM/SRM**. **(A) **General schematic representation of multiple reaction monitoring (MRM). Peptides and glycopeptides from a protease (normally trypsin)-cleaved glycoprotein are subjected to triple quadrupole mass spectrometry (MS). Only selected parent ion ions were selected for fragmentation, and the resulting fragment ion intensities were used for (glyco)peptide quantification. **(B) **Representative chromatogram of simultaneous MRMs of 25 pyridyl amineated sialoglycopeptides found on 16 glycoproteins in mouse serum. Adapted and modified from Kurogochi *et al*. [109].

The true potential of MS in the quantification of protein glycosylation lies in the measurement of total levels of the glycoprotein, while simultaneously measuring its heterogeneously glycosylated subpopulations. The ultimate goal is the development of site-specific label-free methods that are capable of simultaneously quantifying multiple glycopeptides encompassing multiple glycosylation sites and their different glycoforms, using a non-glycosylated peptide from the glycoprotein of interest or a labeled exogenous peptide standard, which could serve as an indicator of the total glycoprotein concentration. Considering that MRM assays have been developed for simultaneous measurement of dozens of tryptic (or other proteolytic) peptides from dozens of proteins, it is not inconceivable that a similar technique could be developed for glycopeptides with different glycan structures from a single, or even multiple proteins. A general schematic of a glycopeptide-targeted MRM from a single glycoprotein can be seen in Figure 3A. However, to improve the sensitivity of such assays, further development and technical advances will be required.

In addition to the general problems with quantifying glycoproteins described above, a number of technical limitations are currently preventing the application of this type of approach to glycoproteins found in samples of clinical interest. The major issue is the much lower ionization efficiency of glycopeptides compared with their non-glycosylated counterparts, generally following the trend that ionization efficiency decreases with glycan branching and sialylation [104,105]. This can result in differences of several orders of magnitude in absolute signal values between glycopeptides and non-glycosylated peptides [104,105]. Additionally, compared with the measurement of non-glycosylated peptides in the same quantity of the protein analyte, the MRM signal for any individual glycopeptide (of which there is a heterogenous population for any given glycosylation site of a glycoprotein) will be significantly lower, because it represents only a subset of a heterogeneous glycoform population. Major complications can also arise in developing a glycopeptide quantification method because of the absence of exogenous glycopeptide standards and incomplete proteolytic digestion cased by steric hinderence by the glycan chains [104,106,107].

Verification of candidate biomarkers in non-serological bio-fluids using MRM/SRM assays has become standard practice in biomarker discovery laboratories. The challenges associated with the development and optimization of MRM assays were significantly eased with the advent of MRM-transition-prediction and data analysis software such as Pinpoint (Thermo Fisher Scientific Inc., Rockford, IL, USA) and Skyline (Open-source software, MacCoss laboratory, University of Washington, Seattle, WA, USA). Owing to the absence of such invaluable tools for glycan-bound peptides, MRM development for this use is still a daunting task. However, the difficulties associated with the prediction of glycopeptide MRM transitions and their optimal collision energies can be overcome by monitoring common oxonium and peptide positive N-acetylhexosamine ions that occur during fragmentation [104,108].

Despite these considerable obstacles, some proof-of-concept studies have been performed. For example, in a recent study by Song *et al*., [104] MRM assays were developed for the quantification of fetuin and alpha1-acid glycoprotein glycopeptides applicable to serum samples. Kurogochi *et al*. have beeen able to develop MRM assays for quantification of 25 glycopeptides from 16 glycoproteins found in serum of mice (Figure 3B) [109]. Specifically, sialic acid moieties on glycopeptide were oxidized with sodium periodate, enriched for by hydrazide chemistry, labeled with 2-aminopyridine, and the resulting labeled sialoglycopeptides were subjected to MS. Preliminary studies have also been performed with purified RNase B and asialofetuin [110]. Haptoglobin glycopeptides were characterized and relatively quantified in serum samples of patients with psoriasis [111] and patients with pancreatic cancer [112]. Ion-current intensities were used to quantify glycopeptides from alpha-1-acid glycoprotein [113]. The core-fucosylated subpopulations of several glycoproteins were quantified using partial deglycosylation with Endo F3 in conjunction with glycopeptide MRMs [114]. With the improvement and evolution of MS technology and sample-preparation techniques, these types of assays will play a more prominent role in the quantification of glycoproteins. In a futuristic scenario, to construct high-throughput platforms for the verification of cancer-exclusive glycoforms, these MRM-MS assays could be coupled to robotic immunoaffinity enrichment methods [115].

## Alternative strategies

Although lectin and MS-based approaches for quantification of glycoproteins are the most common, there are other technologies that are also applied, and new ones are being developed, to be used alone, or in combination with each other. Well-established liquid-chromatography techniques using HILIC or PGC are readily available for enrichment and separation of glycan and glycoconjugates in conjunction with other detection and quantification methods [116-118]. The most established affinity-binding agents for quantification of proteins and other molecules are antibodies, and the ELISA still remains the gold standard for the clinical measurement of serological targets. However, glycan-specific antibodies are extremely rare compared with antibodies recognizing peptide epitopes, and their use in the field is limited compared with lectins. This is because carbohydrates have been shown to be poor immunogens, and their antibodies have affinities comparable with those of lectins, but with a much more difficult development process. In addition, antibodies detecting an epitope that encompasses a part of a given protein's sequence, while at the same time recognizing its glycan structure, thereby giving site and glycoprotein specificity, are extremely rare. Therefore, the possible advantage of using a glycan-specific antibody over a comparable lectin is minor. The issue of cross-reactivity has been brought up for Tn antigen-recognizing antibodies [119]. In a recent study, 27 commonly used carbohydrate-binding antibodies against histo-blood group, Lewis, and tumor antigens were examined for their specificity using a glycan/glycoprotein array [120]. Although some showed high specificity and affinity for their targets, almost half of them exhibited cross-reactivity for other glycan structures. In cancer research, the role of such antibodies has been mostly limited to indirect quantification by immunohistochemistry and blotting. When considering applications of glycan-specific antibodies for serological markers of malignancy, the CA 19-9 and CA 15-3 tests stand out. By using a sandwich ELISA, the CA 19-9 test measures the serum levels of sialyl Lewis^a ^antigen on glycoproteins and glycolipids, and is used for monitoring of pancreatic cancer progression and recurrence, and for differentiation of the cancer from pancreatitis [42,121,122]. The CA 15-3 test is used to quantify a sialylated O-glycosylation epitope on mucin 1 (MUC1), and is used for prognosis and monitoring of treatment for breast cancer [123,124].

Chromatography-based strategies have also been used with some success. Ion-exchange chromatography is being used clinically for separation and quantification of serum transferrin glycoforms to test for congenital disorders of glycosylation [125,126]. KLK6 glycoforms have also been measured in a number of biological fluids, including serum, from patients with ovarian cancer at low concentrations (down to 1 ng/ml) using strong anion exchange for separation and ELISA for quantification [127]. Novel strategies are also being used for the development of new carbohydrate-recognizing agents, which could be used in a quantitative fashion. Phage display technology has been used to improve and alter the binding properties of glycan-binding modules of glycan-processing enzymes and for development of carbohydrate-binding peptides [128-132]. The technique of systematic evolution of ligands by exponential enrichment (SELEX) has been applied to the development of aptamers, single-stranded DNA or RNA oligonucleotides, which have been tried as binding agents for a number of carbohydrate moieties [130,133-137]. The more recent advancements and nascent technologies developed for carbohydrate detection, also referred to as glyco-biosensors, have been reviewed extensively [1,138,139]. Some of these include electrochemical impedance spectroscopy [140-143], molecular 'tweezers' [144], nanoparticle displacement methods [145], quartz crystal microbalance [146,147], and surface plasmon resonance [148-150]. However, these technologies are garnered towards highly controlled *in vitro *systems, and will require further testing before application in a clinical setting.

## Conclusions and perspectives

The clinical potential of glycoprotein biomarkers in cancer is indisputable. Some valuable successes have been achieved in the field, yet there is much room for improvement. The majority of the tools currently available have proven their utility beyond doubt when used for qualitative and characterization purposes. However, for each of these technologies, the leap from analytical to quantitative applications has not been sufficiently successful.

Over the next decade, the major goal will be the reliable detection and quantification of the full scope of glycan heterogeneity on any particular glycoprotein of interest, and the ability to differentiate these patterns between homeostatic and disease conditions. When the recent literature is searched for 'glycosylation quantification', it quickly becomes obvious that MS-based approaches have almost become an absolute requirement. However, when viewing the field as a whole, one gets an appreciation of the fact that MS advancements alone will not bring a major breakthrough. In the near future, the development of even more new glycan-recognition agents can be expected, such as novel naturally occurring or recombinant lectins, carbohydrate-recognizing antibodies, aptamers, and other glycobiosensors. Progress is also being made in the engineering and synthetic production of protein glycosylation, which will greatly aid in the creation of standards and uniform model systems for development of precise quantitative assays. In the near future, the predominance and expansion of immuno-based and lectin-based methods in practical applications of glycan quantification can be expected, especially given the recent advances in microarray technology. We believe that MS holds the greatest potential, but it is still hampered by a number of technical limitations, which will require significant technological progress to be made before it will be sufficiently reliable and applicable in the most appropriate manner. Nonetheless, we believe that MS is the most promising tool for detection and quantification of the full scope of protein-associated glycosylation down to the single monosaccharide unit level. The future appears bright, and progress in the field is inevitable; the only uncertainty is how long it will take.

## Abbreviations

AFP: alpha-fetoprotein; CA: carcinoma antigen; CEA: carcinoembryonic antigen; ELISA: enzyme-linked immunosorbent assay; ELLA: enzyme-linked lectinsorbent assay; hCG: human chorionic gonadotropin; MRM: multiple reaction monitoring; PSA: prostate-specific antigen; PTM: post-translational modification; SDS-PAGE: sodium dodecyl sulfate polyacrylamide gel electrophoresis; SRM: selected reaction monitoring

## Competing interests

The authors declare that they have no competing interests.

## Authors' contributions

UK conceived and wrote the majority of the review. HK assisted in the development of the manuscript and wrote a part of the review dealing with MRM-based quantification of glycopeptides. EPD provided support and supervision throughout the development of the review. All authors have read and approved the manuscript for publication.

## Authors' information

At the time of submission of the manuscript, Uros Kuzmanov was a PhD student in the laboratory of Dr Eleftherios P. Diamandis, Professor of Laboratory Medicine and Pathobiology at the University of Toronto. Dr Hari Kosanam is a postdoctoral fellow in the same laboratory.

## Pre-publication history

The pre-publication history for this paper can be accessed here:

http://www.biomedcentral.com/1741-7015/11/31/prepub

## References

[B1] Cunningham S Gerlach JQ Kane M Joshi L Glyco-biosensors: Recent advances and applications for the detection of free and bound carbohydrates Analyst 2010 135 2471 2480 10.1039/c0an00276c 20714521

[B2] Dalpathado DS Desaire H Glycopeptide analysis by mass spectrometry Analyst 2008 133 731 738 10.1039/b713816d 18493671

[B3] Reinders J Sickmann A Modificomics: posttranslational modifications beyond protein phosphorylation and glycosylation Biomol Eng 2007 24 169 177 10.1016/j.bioeng.2007.03.002 17419095

[B4] Walsh CT Garneau-Tsodikova S Gatto GJ Jr Protein posttranslational modifications: the chemistry of proteome diversifications Angew Chem Int Ed Engl 2005 44 7342 7372 10.1002/anie.200501023 16267872

[B5] Crocker PR Feizi T Carbohydrate recognition systems: functional triads in cell-cell interactions Curr Opin Struct Biol 1996 6 679 691 10.1016/S0959-440X(96)80036-4 8913692

[B6] Feizi T Carbohydrate-mediated recognition systems in innate immunity Immunol Rev 2000 173 79 88 10.1034/j.1600-065X.2000.917310.x 10719669

[B7] Gabius HJ Siebert HC Andre S Jimenez-Barbero J Rudiger H Chemical biology of the sugar code Chembiochem 2004 5 740 764 10.1002/cbic.200300753 15174156

[B8] Helenius A Aebi M Intracellular functions of N-linked glycans Science 2001 291 2364 2369 10.1126/science.291.5512.2364 11269317

[B9] Karlsson KA Meaning and therapeutic potential of microbial recognition of host glycoconjugates Mol Microbiol 1998 29 1 11 10.1046/j.1365-2958.1998.00854.x 9701797

[B10] Apweiler R Hermjakob H Sharon N On the frequency of protein glycosylation, as deduced from analysis of the SWISS-PROT database Biochim Biophys Acta 1999 1473 4 8 10.1016/S0304-4165(99)00165-8 10580125

[B11] Cummings RD The repertoire of glycan determinants in the human glycome Mol Biosyst 2009 5 1087 1104 10.1039/b907931a 19756298

[B12] Schachter H Freeze HH Glycosylation diseases: quo vadis? Biochim Biophys Acta 2009 1792 925 930 10.1016/j.bbadis.2008.11.002 19061954 PMC3927646

[B13] Spiro RG Protein glycosylation: nature, distribution, enzymatic formation, and disease implications of glycopeptide bonds Glycobiology 2002 12 43R 56R 10.1093/glycob/12.4.43R 12042244

[B14] Meezan E Wu HC Black PH Robbins PW Comparative studies on the carbohydrate-containing membrane components of normal and virus-transformed mouse fibroblasts. II. Separation of glycoproteins and glycopeptides by sephadex chromatography Biochemistry 1969 8 2518 2524 10.1021/bi00834a039 4307997

[B15] Wu HC Meezan E Black PH Robbins PW Comparative studies on the carbohydrate-containing membrane components of normal and virus-transformed mouse fibroblasts. I. Glucosamine-labeling patterns in 3T3, spontaneously transformed 3T3, and SV-40-transformed 3T3 cells Biochemistry 1969 8 2509 2517 10.1021/bi00834a038 4307996

[B16] Brooks SA Strategies for analysis of the glycosylation of proteins: current status and future perspectives Mol Biotechnol 2009 43 76 88 10.1007/s12033-009-9184-6 19507069

[B17] Drake PM Cho W Li B Prakobphol A Johansen E Anderson NL Regnier FE Gibson BW Fisher SJ Sweetening the pot: adding glycosylation to the biomarker discovery equation Clin Chem 2010 56 223 236 10.1373/clinchem.2009.136333 19959616 PMC2849286

[B18] Arnold JN Saldova R Hamid UM Rudd PM Evaluation of the serum N-linked glycome for the diagnosis of cancer and chronic inflammation Proteomics 2008 8 3284 3293 10.1002/pmic.200800163 18646009

[B19] Dennis JW Granovsky M Warren CE Glycoprotein glycosylation and cancer progression Biochim Biophys Acta 1999 1473 21 34 10.1016/S0304-4165(99)00167-1 10580127

[B20] Dube DH Bertozzi CR Glycans in cancer and inflammation--potential for therapeutics and diagnostics Nat Rev Drug Discov 2005 4 477 488 10.1038/nrd1751 15931257

[B21] Gorelik E Galili U Raz A On the role of cell surface carbohydrates and their binding proteins (lectins) in tumor metastasis Cancer Metastasis Rev 2001 20 245 277 10.1023/A:1015535427597 12085965

[B22] Hakomori S Glycosylation defining cancer malignancy: new wine in an old bottle Proc Natl Acad Sci USA 2002 99 10231 10233 10.1073/pnas.172380699 12149519 PMC124893

[B23] Granovsky M Fata J Pawling J Muller WJ Khokha R Dennis JW Suppression of tumor growth and metastasis in Mgat5-deficient mice Nat Med 2000 6 306 312 10.1038/73163 10700233

[B24] Guo HB Lee I Kamar M Pierce M N-acetylglucosaminyltransferase V expression levels regulate cadherin-associated homotypic cell-cell adhesion and intracellular signaling pathways J Biol Chem 2003 278 52412 52424 10.1074/jbc.M308837200 14561752

[B25] Handerson T Camp R Harigopal M Rimm D Pawelek J Beta1,6-branched oligosaccharides are increased in lymph node metastases and predict poor outcome in breast carcinoma Clin Cancer Res 2005 11 2969 2973 10.1158/1078-0432.CCR-04-2211 15837749

[B26] Pinho SS Reis CA Paredes J Magalhaes AM Ferreira AC Figueiredo J The role of N-acetylglucosaminyltransferase III and V in the post-transcriptional modifications of E-cadherin Hum Mol Genet 2009 18 2599 2608 10.1093/hmg/ddp194 19403558

[B27] Takahashi M Kuroki Y Ohtsubo K Taniguchi N Core fucose and bisecting GlcNAc, the direct modifiers of the N-glycan core: their functions and target proteins Carbohydr Res 2009 344 1387 1390 10.1016/j.carres.2009.04.031 19508951

[B28] Burchell J Poulsom R Hanby A Whitehouse C Cooper L Clausen H An alpha2,3 sialyltransferase (ST3Gal I) is elevated in primary breast carcinomas Glycobiology 1999 9 1307 1311 10.1093/glycob/9.12.1307 10561455

[B29] Meany D Chan D Aberrant glycosylation associated with enzymes as cancer biomarkers Clinical Proteomics 2011 8 7 10.1186/1559-0275-8-7 21906357 PMC3170274

[B30] Ogawa JI Inoue H Koide S alpha-2,3-Sialyltransferase type 3N and alpha-1,3-fucosyltransferase type VII are related to sialyl Lewis(x) synthesis and patient survival from lung carcinoma Cancer 1997 79 1678 1685 10.1002/(SICI)1097-0142(19970501)79:9<1678::AID-CNCR7>3.0.CO;2-8 9128982

[B31] Petretti T Kemmner W Schulze B Schlag PM Altered mRNA expression of glycosyltransferases in human colorectal carcinomas and liver metastases Gut 2000 46 359 366 10.1136/gut.46.3.359 10673297 PMC1727852

[B32] Picco G Julien S Brockhausen I Beatson R Antonopoulos A Haslam S Over-expression of ST3Gal-I promotes mammary tumorigenesis Glycobiology 2010 20 1241 1250 10.1093/glycob/cwq085 20534593 PMC2934706

[B33] Recchi MA Hebbar M Hornez L Harduin-Lepers A Peyrat JP Delannoy P Multiplex reverse transcription polymerase chain reaction assessment of sialyltransferase expression in human breast cancer Cancer Res 1998 58 4066 4070 9751611

[B34] Fukushima K Satoh T Baba S Yamashita K alpha1,2-Fucosylated and beta-N-acetylgalactosaminylated prostate-specific antigen as an efficient marker of prostatic cancer Glycobiology 2010 20 452 460 10.1093/glycob/cwp197 20008118

[B35] Jankovic MM Milutinovic BS Glycoforms of CA125 antigen as a possible cancer marker Cancer Biomark 2008 4 35 42 18334732 10.3233/cbm-2008-4104

[B36] Valmu L Alfthan H Hotakainen K Birken S Stenman UH Site-specific glycan analysis of human chorionic gonadotropin beta-subunit from malignancies and pregnancy by liquid chromatography--electrospray mass spectrometry Glycobiology 2006 16 1207 1218 10.1093/glycob/cwl034 16880503

[B37] Saeland E Belo AI Mongera S van Die I Meijer GA van Kooyk Y Differential glycosylation of MUC1 and CEACAM5 between normal mucosa and tumour tissue of colon cancer patients Int J Cancer 2012 131 117 128 10.1002/ijc.26354 21823122

[B38] Narimatsu H Sawaki H Kuno A Kaji H Ito H Ikehara Y A strategy for discovery of cancer glyco-biomarkers in serum using newly developed technologies for glycoproteomics FEBS J 2010 277 95 105 10.1111/j.1742-4658.2009.07430.x 19919546

[B39] Pan S Chen R Aebersold R Brentnall TA Mass spectrometry based glycoproteomics - from a proteomics perspective Mol Cell Proteomics 2011 10 10.1074/mcp.R110.003251 PMC3013464 20736408

[B40] Hua S An HJ Glycoscience aids in biomarker discovery BMB Rep 2012 45 323 330 10.5483/BMBRep.2012.45.6.132 22732216

[B41] Adamczyk B Tharmalingam T Rudd PM Glycans as cancer biomarkers Biochim Biophys Acta 2011 10.1016/j.bbagen.2011.12.001 22178561

[B42] Goonetilleke KS Siriwardena AK Systematic review of carbohydrate antigen (CA 19-9) as a biochemical marker in the diagnosis of pancreatic cancer Eur J Surg Oncol 2007 33 266 270 10.1016/j.ejso.2006.10.004 17097848

[B43] Donati M Brancato G Donati A Clinical biomarkers in hepatocellular carcinoma (HCC) Front Biosci (Schol Ed) 2010 2 571 577 20036969 10.2741/s86

[B44] Li D Mallory T Satomura S AFP-L3: a new generation of tumor marker for hepatocellular carcinoma Clin Chim Acta 2001 313 15 19 10.1016/S0009-8981(01)00644-1 11694234

[B45] Gamblin DP Scanlan EM Davis BG Glycoprotein synthesis: an update Chem Rev 2009 109 131 163 10.1021/cr078291i 19093879

[B46] Peracaula R Tabares G Royle L Harvey DJ Dwek RA Rudd PM de Llorens R Altered glycosylation pattern allows the distinction between prostate-specific antigen (PSA) from normal and tumor origins Glycobiology 2003 13 457 470 10.1093/glycob/cwg041 12626390

[B47] Anderson NL Anderson NG The human plasma proteome: history, character, and diagnostic prospects Mol Cell Proteomics 2002 1 845 867 10.1074/mcp.R200007-MCP200 12488461

[B48] Sharon N Lis H History of lectins: from hemagglutinins to biological recognition molecules Glycobiology 2004 14 53R 62R 10.1093/glycob/cwh122 15229195

[B49] Bies C Lehr CM Woodley JF Lectin-mediated drug targeting: history and applications Adv Drug Deliv Rev 2004 56 425 435 10.1016/j.addr.2003.10.030 14969751

[B50] Sharon N Lis H Lectins: cell-agglutinating and sugar-specific proteins Science 1972 177 949 959 10.1126/science.177.4053.949 5055944

[B51] McCoy JP Jr Varani J Goldstein IJ Enzyme-linked lectin assay (ELLA): use of alkaline phosphatase-conjugated Griffonia simplicifolia B4 isolectin for the detection of alpha-D-galactopyranosyl end groups Anal Biochem 1983 130 437 444 10.1016/0003-2697(83)90613-9 6869832

[B52] McCoy JP Jr Goldstein IJ Varani J A review of studies in our laboratory regarding ELLA methodology for the study of cell surface carbohydrates from tumors of varying metastatic potential Tumour Biol 1985 6 99 114 3901226

[B53] Reddi AL Sankaranarayanan K Arulraj HS Devaraj N Devaraj H Enzyme-linked PNA lectin-binding assay of serum T-antigen in patients with SCC of the uterine cervix Cancer Lett 2000 149 207 211 10.1016/S0304-3835(99)00363-8 10737726

[B54] Zhang SL Liang YR Li JL Dai YR Huang D Preliminary studies of serum glycoconjugates in patients with cancer using the enzyme-linked lectin assay Cancer Biochem Biophys 1990 11 211 216 2268850

[B55] Graham K Keller K Ezzell J Doyle R Enzyme-linked lectinosorbent assay (ELLA) for detecting Bacillus anthracis Eur J Clin Microbiol 1984 3 210 212 10.1007/BF02014881 6432529

[B56] Leriche V Sibille P Carpentier B Use of an enzyme-linked lectinsorbent assay to monitor the shift in polysaccharide composition in bacterial biofilms Appl Environ Microbiol 2000 66 1851 1856 10.1128/AEM.66.5.1851-1856.2000 10788349 PMC101422

[B57] Thomas VL Sanford BA Moreno R Ramsay MA Enzyme-linked lectinsorbent assay measures N-acetyl-D-glucosamine in matrix of biofilm produced by Staphylococcus epidermidis Curr Microbiol 1997 35 249 254 10.1007/s002849900248 9290068

[B58] Parker N Makin CA Ching CK Eccleston D Taylor OM Milton JD Rhodes JM A new enzyme-linked lectin/mucin antibody sandwich assay (CAM 17.1/WGA) assessed in combination with CA 19-9 and peanut lectin binding assay for the diagnosis of pancreatic cancer Cancer 1992 70 1062 1068 10.1002/1097-0142(19920901)70:5<1062::AID-CNCR2820700509>3.0.CO;2-P 1515982

[B59] Gornik O Lauc G Enzyme linked lectin assay (ELLA) for direct analysis of transferrin sialylation in serum samples Clin Biochem 2007 40 718 723 10.1016/j.clinbiochem.2007.01.010 17320850

[B60] Dwek MV Jenks A Leathem AJ A sensitive assay to measure biomarker glycosylation demonstrates increased fucosylation of prostate specific antigen (PSA) in patients with prostate cancer compared with benign prostatic hyperplasia Clin Chim Acta 2010 411 1935 1939 10.1016/j.cca.2010.08.009 20708609

[B61] Kim HJ Lee SJ Antibody-based enzyme-linked lectin assay (ABELLA) for the sialylated recombinant human erythropoietin present in culture supernatant J Pharm Biomed Anal 2008 48 716 721 10.1016/j.jpba.2008.07.004 18722069

[B62] Cook DB Bustamam AA Brotherick I Shenton BK Self CH Lectin ELISA for the c-erb-B2 tumor marker protein p185 in patients with breast cancer and controls Clin Chem 1999 45 292 295 9931057

[B63] Matsumoto H Shinzaki S Narisada M Kawamoto S Kuwamoto K Moriwaki K Kanke F Satomura S Kumada T Miyoshi E Clinical application of a lectin-antibody ELISA to measure fucosylated haptoglobin in sera of patients with pancreatic cancer Clin Chem Lab Med 2010 48 505 512 20128732 10.1515/CCLM.2010.095

[B64] Chen S Haab BB Analysis of glycans on serum proteins using antibody microarrays Methods Mol Biol 2009 520 39 58 10.1007/978-1-60327-811-9_4 19381946 PMC3772347

[B65] Li C Lubman DM Analysis of serum protein glycosylation with antibody-lectin microarray for high-throughput biomarker screening Methods Mol Biol 2011 723 15 28 10.1007/978-1-61779-043-0_2 21370056

[B66] Thompson R Creavin A O'Connell M O'Connor B Clarke P Optimization of the enzyme-linked lectin assay for enhanced glycoprotein and glycoconjugate analysis Anal Biochem 2011 413 114 122 10.1016/j.ab.2011.02.013 21320462

[B67] Meany DL Sokoll LJ Chan DW Early Detection of Cancer: Immunoassays for Plasma Tumor Markers Expert Opin Med Diagn 2009 3 597 605 10.1517/17530050903266830 19966928 PMC2788950

[B68] Yoshida S Kurokohchi K Arima K Masaki T Hosomi N Funaki T Murota M Kita Y Watanabe S Kuriyama S Clinical significance of lens culinaris agglutinin-reactive fraction of serum alpha-fetoprotein in patients with hepatocellular carcinoma Int J Oncol 2002 20 305 309 11788893

[B69] Johnson PJ Poon TC Hjelm NM Ho CS Ho SK Welby C Stevenson D Patel T Parekh R Townsend RR Glycan composition of serum alpha-fetoprotein in patients with hepatocellular carcinoma and non-seminomatous germ cell tumour Br J Cancer 1999 81 1188 1195 10.1038/sj.bjc.6690828 10584881 PMC2374329

[B70] Nakagawa T Miyoshi E Yakushijin T Hiramatsu N Igura T Hayashi N Taniguchi N Kondo A Glycomic analysis of alpha-fetoprotein L3 in hepatoma cell lines and hepatocellular carcinoma patients J Proteome Res 2008 7 2222 2233 10.1021/pr700841q 18479159

[B71] Haab BB Yue T High-throughput studies of protein glycoforms using antibody-lectin sandwich arrays Methods Mol Biol 2011 785 223 236 10.1007/978-1-61779-286-1_15 21901603 PMC3705222

[B72] Katrlik J Svitel J Gemeiner P Kozar T Tkac J Glycan and lectin microarrays for glycomics and medicinal applications Med Res Rev 2010 30 394 418 20099267 10.1002/med.20195

[B73] Patwa T Li C Simeone DM Lubman DM Glycoprotein analysis using protein microarrays and mass spectrometry Mass Spectrom Rev 2010 29 830 844 10.1002/mas.20269 20077480 PMC2889184

[B74] Haab BB Porter A Yue T Li L Scheiman J Anderson MA Barnes D Schmidt CM Feng Z Simeone DM Glycosylation variants of mucins and CEACAMs as candidate biomarkers for the diagnosis of pancreatic cystic neoplasms Ann Surg 2010 251 937 945 10.1097/SLA.0b013e3181d7738d 20395854 PMC3713623

[B75] Li C Simeone DM Brenner DE Anderson MA Shedden KA Ruffin MT Lubman DM Pancreatic cancer serum detection using a lectin/glyco-antibody array method J Proteome Res 2009 8 483 492 10.1021/pr8007013 19072160 PMC2637303

[B76] Yue T Goldstein IJ Hollingsworth MA Kaul K Brand RE Haab BB The prevalence and nature of glycan alterations on specific proteins in pancreatic cancer patients revealed using antibody-lectin sandwich arrays Mol Cell Proteomics 2009 8 1697 1707 10.1074/mcp.M900135-MCP200 19377061 PMC2709194

[B77] Hart GW Copeland RJ Glycomics hits the big time Cell 2010 143 672 676 10.1016/j.cell.2010.11.008 21111227 PMC3008369

[B78] Meany DL Zhang Z Sokoll LJ Zhang H Chan DW Glycoproteomics for prostate cancer detection: changes in serum PSA glycosylation patterns J Proteome Res 2009 8 613 619 10.1021/pr8007539 19035787 PMC2997339

[B79] Arnold JN Saldova R Galligan MC Murphy TB Mimura-Kimura Y Telford JE Godwin AK Rudd PM Novel glycan biomarkers for the detection of lung cancer J Proteome Res 2011 10 1755 1764 10.1021/pr101034t 21214223

[B80] Bones J Mittermayr S O'Donoghue N Guttman A Rudd PM Ultra performance liquid chromatographic profiling of serum N-glycans for fast and efficient identification of cancer associated alterations in glycosylation Anal Chem 2010 82 10208 10215 10.1021/ac102860w 21073175

[B81] Zauner G Koeleman CA Deelder AM Wuhrer M Protein glycosylation analysis by HILIC-LC-MS of proteinase K-generated N- and O-glycopeptides J Sep Sci 2010 33 903 910 10.1002/jssc.200900850 20222081

[B82] Li B An HJ Kirmiz C Lebrilla CB Lam KS Miyamoto S Glycoproteomic analyses of ovarian cancer cell lines and sera from ovarian cancer patients show distinct glycosylation changes in individual proteins J Proteome Res 2008 7 3776 3788 10.1021/pr800297u 18642944

[B83] Ruhaak LR Miyamoto S Kelly K Lebrilla CB N-Glycan profiling of dried blood spots Anal Chem 2012 84 396 402 10.1021/ac202775t 22128873 PMC3259271

[B84] Alley WR Jr Madera M Mechref Y Novotny MV Chip-based reversed-phase liquid chromatography-mass spectrometry of permethylated N-linked glycans: a potential methodology for cancer-biomarker discovery Anal Chem 2011 82 5095 5106 20491449 10.1021/ac100131e PMC2910595

[B85] Mechref Y Analysis of glycans derived from glycoconjugates by capillary electrophoresis-mass spectrometry Electrophoresis 2011 32 3467 3481 10.1002/elps.201100342 22180203 PMC3360420

[B86] Kita Y Miura Y Furukawa J Nakano M Shinohara Y Ohno M Takimoto A Nishimura S Quantitative glycomics of human whole serum glycoproteins based on the standardized protocol for liberating N-glycans Mol Cell Proteomics 2007 6 1437 1445 10.1074/mcp.T600063-MCP200 17522412

[B87] Kyselova Z Mechref Y Kang P Goetz JA Dobrolecki LE Sledge GW Schnaper L Hickey RJ Malkas LH Novotny MV Breast cancer diagnosis and prognosis through quantitative measurements of serum glycan profiles Clin Chem 2008 54 1166 1175 10.1373/clinchem.2007.087148 18487288

[B88] Imre T Kremmer T Heberger K Molnar-Szollosi E Ludanyi K Pocsfalvi G Malorni A Drahos L Vekey K Mass spectrometric and linear discriminant analysis of N-glycans of human serum alpha-1-acid glycoprotein in cancer patients and healthy individuals J Proteomics 2008 71 186 197 10.1016/j.jprot.2008.04.005 18617146

[B89] Tsai HY Boonyapranai K Sriyam S Yu CJ Wu SW Khoo KH Phutrakul S Chen ST Glycoproteomics analysis to identify a glycoform on haptoglobin associated with lung cancer Proteomics 2011 11 2162 2170 10.1002/pmic.201000319 21538882

[B90] Zhang S Shu H Luo K Kang X Zhang Y Lu H Liu Y N-linked glycan changes of serum haptoglobin beta chain in liver disease patients Mol Biosyst 2011 7 1621 1628 10.1039/c1mb05020f 21380457

[B91] Drabovich AP Diamandis EP Combinatorial peptide libraries facilitate development of multiple reaction monitoring assays for low-abundance proteins J Proteome Res 2010 9 1236 1245 10.1021/pr900729g 20070123

[B92] Keshishian H Addona T Burgess M Kuhn E Carr SA Quantitative, multiplexed assays for low abundance proteins in plasma by targeted mass spectrometry and stable isotope dilution Mol Cell Proteomics 2007 6 2212 2229 10.1074/mcp.M700354-MCP200 17939991 PMC2435059

[B93] Keshishian H Addona T Burgess M Mani DR Shi X Kuhn E Sabatine MS Gerszten RE Carr SA Quantification of cardiovascular biomarkers in patient plasma by targeted mass spectrometry and stable isotope dilution Mol Cell Proteomics 2009 8 2339 2349 10.1074/mcp.M900140-MCP200 19596694 PMC2758760

[B94] Kulasingam V Smith CR Batruch I Buckler A Jeffery DA Diamandis EP "Product ion monitoring" assay for prostate-specific antigen in serum using a linear ion-trap J Proteome Res 2008 7 640 647 10.1021/pr7005999 18186600

[B95] Anderson NL Anderson NG Haines LR Hardie DB Olafson RW Pearson TW Mass spectrometric quantitation of peptides and proteins using stable isotope standards and capture by anti-peptide antibodies (SISCAPA) J Proteome Res 2004 3 235 244 10.1021/pr034086h 15113099

[B96] Anderson NL Jackson A Smith D Hardie D Borchers C Pearson TW SISCAPA peptide enrichment on magnetic beads using an in-line bead trap device Mol Cell Proteomics 2009 8 995 1005 10.1074/mcp.M800446-MCP200 19196707 PMC2689780

[B97] Kulasingam V Smith CR Batruch I Diamandis EP Immuno-mass spectrometry: quantification of low-abundance proteins in biological fluids Methods Mol Biol 2011 728 207 218 10.1007/978-1-61779-068-3_13 21468950

[B98] Whiteaker JR Zhao L Anderson L Paulovich AG An automated and multiplexed method for high throughput peptide immunoaffinity enrichment and multiple reaction monitoring mass spectrometry-based quantification of protein biomarkers Mol Cell Proteomics 2010 9 184 196 10.1074/mcp.M900254-MCP200 19843560 PMC2808264

[B99] Zhao L Whiteaker JR Pope ME Kuhn E Jackson A Anderson NL Pearson TW Carr SA Paulovich AG Quantification of proteins using peptide immunoaffinity enrichment coupled with mass spectrometry J Vis Exp 2011 10.3791/2812 PMC3197439 21841765

[B100] Ahn YH Kim YS Ji ES Lee JY Jung JA Ko JH Yoo JS Comparative quantitation of aberrant glycoforms by lectin-based glycoprotein enrichment coupled with multiple-reaction monitoring mass spectrometry Anal Chem 2010 82 4441 4447 10.1021/ac1001965 20462175

[B101] Ahn YH Lee JY Kim YS Ko JH Yoo JS Quantitative analysis of an aberrant glycoform of TIMP1 from colon cancer serum by L-PHA-enrichment and SISCAPA with MRM mass spectrometry J Proteome Res 2009 8 4216 4224 10.1021/pr900269s 19645485

[B102] Ahn YH Shin PM Oh NR Park GW Kim H Yoo JS A lectin-coupled, targeted proteomic mass spectrometry (MRM MS) platform for identification of multiple liver cancer biomarkers in human plasma J Proteomics 2012 75 5507 5515 10.1016/j.jprot.2012.06.027 22789673

[B103] Li Y Tian Y Rezai T Prakash A Lopez MF Chan DW Zhang H Simultaneous analysis of glycosylated and sialylated prostate-specific antigen revealing differential distribution of glycosylated prostate-specific antigen isoforms in prostate cancer tissues Anal Chem 2011 83 240 245 10.1021/ac102319g 21141837 PMC3031300

[B104] Song E Pyreddy S Mechref Y Quantification of glycopeptides by multiple reaction monitoring liquid chromatography/tandem mass spectrometry Rapid Commun Mass Spectrom 2012 26 1941 1954 10.1002/rcm.6290 22847692 PMC3673029

[B105] Wada Y Azadi P Costello CE Dell A Dwek RA Geyer H Geyer R Kakehi K Karlsson NG Kato K Kawasaki N Khoo KH Kim S Kondo A Lattova E Mechref Y Miyoshi E Nakamura K Narimatsu H Novotny MV Packer NH Perreault H Peter-Katalinic J Pohlentz G Reinhold VN Rudd PM Suzuki A Taniguchi N Comparison of the methods for profiling glycoprotein glycans--HUPO Human Disease Glycomics/Proteome Initiative multi-institutional study Glycobiology 2007 17 411 422 10.1093/glycob/cwl086 17223647

[B106] Chen R Jiang X Sun D Han G Wang F Ye M Wang L Zou H Glycoproteomics analysis of human liver tissue by combination of multiple enzyme digestion and hydrazide chemistry J Proteome Res 2009 8 651 661 10.1021/pr8008012 19159218

[B107] Cutalo JM Deterding LJ Tomer KB Characterization of glycopeptides from HIV-I(SF2) gp120 by liquid chromatography mass spectrometry J Am Soc Mass Spectrom 2004 15 1545 1555 10.1016/j.jasms.2004.07.008 15519221 PMC1351241

[B108] Leymarie N Zaia J Effective use of mass spectrometry for glycan and glycopeptide structural analysis Anal Chem 2012 84 3040 3048 10.1021/ac3000573 22360375 PMC3319649

[B109] Kurogochi M Matsushista T Amano M Furukawa J Shinohara Y Aoshima M Nishimura S Sialic acid-focused quantitative mouse serum glycoproteomics by multiple reaction monitoring assay Mol Cell Proteomics 2010 9 2354 2368 10.1074/mcp.M110.000430 20571061 PMC2984228

[B110] Rebecchi KR Wenke JL Go EP Desaire H Label-free quantitation: a new glycoproteomics approach J Am Soc Mass Spectrom 2009 20 1048 1059 10.1016/j.jasms.2009.01.013 19278867

[B111] Maresca B Cigliano L Corsaro MM Pieretti G Natale M Bucci EM Dal Piaz F Balato N Nino M Ayala F Abrescia P Quantitative determination of haptoglobin glycoform variants in psoriasis Biol Chem 2010 391 1429 1439 21087091 10.1515/BC.2010.146

[B112] Nakano M Nakagawa T Ito T Kitada T Hijioka T Kasahara A Tajiri M Wada Y Taniguchi N Miyoshi E Site-specific analysis of N-glycans on haptoglobin in sera of patients with pancreatic cancer: a novel approach for the development of tumor markers Int J Cancer 2008 122 2301 2309 10.1002/ijc.23364 18214858

[B113] Ivancic MM Gadgil HS Halsall HB Treuheit MJ LC/MS analysis of complex multiglycosylated human alpha(1)-acid glycoprotein as a model for developing identification and quantitation methods for intact glycopeptide analysis Anal Biochem 2010 400 25 32 10.1016/j.ab.2010.01.026 20100450

[B114] Zhao Y Jia W Wang J Ying W Zhang Y Qian X Fragmentation and site-specific quantification of core fucosylated glycoprotein by multiple reaction monitoring-mass spectrometry Anal Chem 2011 83 8802 8809 10.1021/ac201676a 21970473

[B115] Lopez MF Rezai T Sarracino DA Prakash A Krastins B Athanas M Singh RJ Barnidge DR Oran P Borges C Nelson RW Selected reaction monitoring-mass spectrometric immunoassay responsive to parathyroid hormone and related variants Clin Chem 2010 56 281 290 10.1373/clinchem.2009.137323 20022981

[B116] Hua S Lebrilla C An HJ Application of nano-LC-based glycomics towards biomarker discovery Bioanalysis 2011 3 2573 2585 10.4155/bio.11.263 22122604

[B117] Ikegami T Tomomatsu K Takubo H Horie K Tanaka N Separation efficiencies in hydrophilic interaction chromatography J Chromatogr A 2008 1184 474 503 10.1016/j.chroma.2008.01.075 18294645

[B118] Zauner G Deelder AM Wuhrer M Recent advances in hydrophilic interaction liquid chromatography (HILIC) for structural glycomics Electrophoresis 2011 32 3456 3466 10.1002/elps.201100247 22180202

[B119] Manimala JC Li Z Jain A VedBrat S Gildersleeve JC Carbohydrate array analysis of anti-Tn antibodies and lectins reveals unexpected specificities: implications for diagnostic and vaccine development Chembiochem 2005 6 2229 2241 10.1002/cbic.200500165 16252298

[B120] Manimala JC Roach TA Li Z Gildersleeve JC High-throughput carbohydrate microarray profiling of 27 antibodies demonstrates widespread specificity problems Glycobiology 2007 17 17C 23C 10.1093/glycob/cwm047 17483136

[B121] Magnani JL Nilsson B Brockhaus M Zopf D Steplewski Z Koprowski H Ginsburg V A monoclonal antibody-defined antigen associated with gastrointestinal cancer is a ganglioside containing sialylated lacto-N-fucopentaose II J Biol Chem 1982 257 14365 14369 7142214

[B122] Safi F Schlosser W Kolb G Beger HG Diagnostic value of CA 19-9 in patients with pancreatic cancer and nonspecific gastrointestinal symptoms J Gastrointest Surg 1997 1 106 112 10.1016/S1091-255X(97)80097-2 9834336

[B123] Duffy MJ Evoy D McDermott EW CA 15-3: uses and limitation as a biomarker for breast cancer Clin Chim Acta 2010 411 1869 1874 10.1016/j.cca.2010.08.039 20816948

[B124] von Mensdorff-Pouilly S Gourevitch MM Kenemans P Verstraeten AA van Kamp GJ Kok A van Uffelen K Snijdewint FG Paul MA Meijer S Hilgers J An enzyme-linked immunosorbent assay for the measurement of circulating antibodies to polymorphic epithelial mucin (MUC1) Tumour Biol 1998 19 186 195 10.1159/000030006 9591045

[B125] Babovic-Vuksanovic D O'Brien JF Laboratory diagnosis of congenital disorders of glycosylation type I by analysis of transferrin glycoforms Mol Diagn Ther 2007 11 303 311 10.1007/BF03256251 17963418

[B126] Helander A Bergstrom J Freeze HH Testing for congenital disorders of glycosylation by HPLC measurement of serum transferrin glycoforms Clin Chem 2004 50 954 958 10.1373/clinchem.2003.029629 15105360

[B127] Kuzmanov U Smith CR Batruch I Soosaipillai A Diamandis A Diamandis EP Separation of kallikrein 6 glycoprotein subpopulations in biological fluids by anion-exchange chromatography coupled to ELISA and identification by mass spectrometry Proteomics 2012 12 799 809 10.1002/pmic.201100371 22539431

[B128] Gullfot F Tan TC von Schantz L Karlsson EN Ohlin M Brumer H Divne C The crystal structure of XG-34, an evolved xyloglucan-specific carbohydrate-binding module Proteins 2010 78 785 789 19950365 10.1002/prot.22642

[B129] Gunnarsson LC Dexlin L Karlsson EN Holst O Ohlin M Evolution of a carbohydrate binding module into a protein-specific binder Biomol Eng 2006 23 111 117 10.1016/j.bioeng.2005.12.002 16427804

[B130] Matsubara T Iida M Tsumuraya T Fujii I Sato T Selection of a carbohydrate-binding domain with a helix-loop-helix structure Biochemistry 2008 47 6745 6751 10.1021/bi8000837 18540680

[B131] Matsubara T Sumi M Kubota H Taki T Okahata Y Sato T Inhibition of influenza virus infections by sialylgalactose-binding peptides selected from a phage library J Med Chem 2009 52 4247 4256 10.1021/jm801570y 19558186

[B132] von Schantz L Gullfot F Scheer S Filonova L Cicortas Gunnarsson L Flint JE Daniel G Nordberg-Karlsson E Brumer H Ohlin M Affinity maturation generates greatly improved xyloglucan-specific carbohydrate binding modules BMC Biotechnol 2009 9 92 10.1186/1472-6750-9-92 19878581 PMC2783032

[B133] Boese BJ Breaker RR In vitro selection and characterization of cellulose-binding DNA aptamers Nucleic Acids Res 2007 35 6378 6388 10.1093/nar/gkm708 17881365 PMC2095800

[B134] Boese BJ Corbino K Breaker RR In vitro selection and characterization of cellulose-binding RNA aptamers using isothermal amplification Nucleosides Nucleotides Nucleic Acids 2008 27 949 966 10.1080/15257770802257903 18696364 PMC5360192

[B135] Ferreira CS Cheung MC Missailidis S Bisland S Gariepy J Phototoxic aptamers selectively enter and kill epithelial cancer cells Nucleic Acids Res 2009 37 866 876 10.1093/nar/gkn967 19103663 PMC2647295

[B136] Li M Lin N Huang Z Du L Altier C Fang H Wang B Selecting aptamers for a glycoprotein through the incorporation of the boronic acid moiety J Am Chem Soc 2008 130 12636 12638 10.1021/ja801510d 18763762 PMC2655352

[B137] Rose CM Hayes MJ Stettler GR Hickey SF Axelrod TM Giustini NP Suljak SW Capillary electrophoretic development of aptamers for a glycosylated VEGF peptide fragment Analyst 2010 135 2945 2951 10.1039/c0an00445f 20820497 PMC2957546

[B138] Gerlach JQ Cunningham S Kane M Joshi L Glycobiomimics and glycobiosensors Biochem Soc Trans 2010 38 1333 1336 10.1042/BST0381333 20863309

[B139] Jelinek R Kolusheva S Carbohydrate biosensors Chem Rev 2004 104 5987 6015 10.1021/cr0300284 15584694

[B140] Cheng W Ding L Lei J Ding S Ju H Effective cell capture with tetrapeptide-functionalized carbon nanotubes and dual signal amplification for cytosensing and evaluation of cell surface carbohydrate Anal Chem 2008 80 3867 3872 10.1021/ac800199t 18407618

[B141] Ding L Cheng W Wang X Ding S Ju H Carbohydrate monolayer strategy for electrochemical assay of cell surface carbohydrate J Am Chem Soc 2008 130 7224 7225 10.1021/ja801468b 18489098

[B142] La Belle JT Gerlach JQ Svarovsky S Joshi L Label-free impedimetric detection of glycan-lectin interactions Anal Chem 2007 79 6959 6964 10.1021/ac070651e 17658764

[B143] Oliveira MD Correia MT Diniz FB Concanavalin A and polyvinyl butyral use as a potential dengue electrochemical biosensor Biosens Bioelectron 2009 25 728 732 10.1016/j.bios.2009.08.009 19747814

[B144] Phillips MD Fyles TM Barwell NP James TD Carbohydrate sensing using a fluorescent molecular tweezer Chem Commun (Camb) 2009 6557 6559 19865648 10.1039/b909230g

[B145] Dai Z Kawde AN Xiang Y La Belle JT Gerlach J Bhavanandan VP Joshi L Wang J Nanoparticle-based sensing of glycan-lectin interactions J Am Chem Soc 2006 128 10018 10019 10.1021/ja063565p 16881623

[B146] Pei Z Anderson H Aastrup T Ramstrom O Study of real-time lectin-carbohydrate interactions on the surface of a quartz crystal microbalance Biosens Bioelectron 2005 21 60 66 10.1016/j.bios.2004.10.006 15967351

[B147] Shen Z Huang M Xiao C Zhang Y Zeng X Wang PG Nonlabeled quartz crystal microbalance biosensor for bacterial detection using carbohydrate and lectin recognitions Anal Chem 2007 79 2312 2319 10.1021/ac061986j 17295446 PMC2519234

[B148] Astrom E Ohlson S Detection of weakly interacting anti-carbohydrate scFv phages using surface plasmon resonance J Mol Recognit 2006 19 282 286 10.1002/jmr.786 16739238

[B149] Foley KJ Forzani ES Joshi L Tao N Detection of lectin-glycan interaction using high resolution surface plasmon resonance Analyst 2008 133 744 746 10.1039/b719321a 18493673

[B150] Mercey E Sadir R Maillart E Roget A Baleux F Lortat-Jacob H Livache T Polypyrrole oligosaccharide array and surface plasmon resonance imaging for the measurement of glycosaminoglycan binding interactions Anal Chem 2008 80 3476 3482 10.1021/ac800226k 18348577

[B151] Wu C Orozco C Boyer J Leglise M Goodale J Batalov S Hodge CL Haase J Janes J Huss JW Su AI BioGPS: an extensible and customizable portal for querying and organizing gene annotation resources Genome Biol 2009 10 R130 10.1186/gb-2009-10-11-r130 19919682 PMC3091323

[B152] Su AI Wiltshire T Batalov S Lapp H Ching KA Block D Zhang J Soden R Hayakawa M Kreiman G Cooke MP Walker JR Hogenesch JB A gene atlas of the mouse and human protein-encoding transcriptomes Proc Natl Acad Sci USA 2004 101 6062 6067 10.1073/pnas.0400782101 15075390 PMC395923

[B153] Abelev GI Production of embryonal alpha-globulin by transplantable mouse hepatomas Transplantation 1963 1 174 180 10.1097/00007890-196301020-00004 14010646

[B154] Tumor markers: physiology, pathobiology, technology, and clinical applications 2002 Washington, DC: AACC Press

[B155] Bagshawe KD Markers in gynaecological cancer Arch Gynecol 1980 229 303 310 10.1007/BF02108581 7191243

[B156] Bast RC Reactivity of a monoclonal antibody with human ovarian carcinoma J Clin Invest 1981 68 1331 1337 10.1172/JCI110380 7028788 PMC370929

[B157] Bast RC 2000 update of recommendations for the use of tumor markers in breast and colorectal cancer: clinical practice guidelines of the American Society of Clinical Oncology J Clin Oncol 2001 19 1865 1878 11251019 10.1200/JCO.2001.19.6.1865

[B158] Hilkens J Monoclonal antibodies against human milk-fat globule membranes detecting differentiation antigens of the mammary gland and its tumors Int J Cancer 1984 34 197 206 10.1002/ijc.2910340210 6206003

[B159] Kufe D Differential reactivity of a novel monoclonal antibody (DF3) with human malignant versus benign breast tumors Hybridoma 1984 3 223 232 10.1089/hyb.1984.3.223 6094338

[B160] Koprowski H Colorectal carcinoma antigens detected by hybridoma antibodies Somatic Cell Genet 1979 5 957 971 10.1007/BF01542654 94699

[B161] Ludwig JA Weinstein JN Biomarkers in cancer staging, prognosis and treatment selection Nat Rev Cancer 2005 5 845 856 10.1038/nrc1739 16239904

[B162] Coussens L Tyrosine kinase receptor with extensive homology to EGF receptor shares chromosomal location with neu oncogene Science 1985 230 1132 1139 10.1126/science.2999974 2999974

[B163] Yamamoto T Similarity of protein encoded by the human c-erb-B-2 gene to epidermal growth factor receptor Nature 1986 319 230 234 10.1038/319230a0 3003577

[B164] Wang MC Purification of a human prostate specific antigen Invest Urol 1979 17 159 163 89106

[B165] Carayanniotis G Rao VP Searching for pathogenic epitopes in thyroglobulin: parameters and caveats Immunol Today 1977 18 83 88 9057359 10.1016/s0167-5699(96)10073-6

[B166] Sturgeon C Practice guidelines for tumor marker use in the clinic Clin Chem 2002 48 1151 1159 12142367

[B167] Beveridge RA Review of clinical studies of CA 27.29 in breast cancer management Int J Biol Markers 1999 14 36 39 10367248 10.1177/172460089901400107

[B168] Kulasingam V Diamandis EP Strategies for discovering novel cancer biomarkers through utilization of emerging technologies Nat Clin Pract Oncol 2008 5 588 599 18695711 10.1038/ncponc1187

